# Dysregulation of the Mitochondrial Proteome Occurs in Mice Lacking Adiponectin Receptor 1

**DOI:** 10.3389/fendo.2019.00872

**Published:** 2019-12-13

**Authors:** Mark E. Pepin, Christoph Koentges, Katharina Pfeil, Johannes Gollmer, Sophia Kersting, Sebastian Wiese, Michael M. Hoffmann, Katja E. Odening, Constantin von zur Mühlen, Philipp Diehl, Peter Stachon, Dennis Wolf, Adam R. Wende, Christoph Bode, Andreas Zirlik, Heiko Bugger

**Affiliations:** ^1^Division of Molecular and Cellular Pathology, Department of Pathology, University of Alabama at Birmingham, Birmingham, AL, United States; ^2^Division of Cardiology and Angiology I, Heart Center Freiburg University, Freiburg, Germany; ^3^Division of Cardiology, Medical University of Graz, Graz, Austria; ^4^Core Unit Mass Spectrometry and Proteomics, Ulm University, Ulm, Germany; ^5^Institute for Clinical Chemistry and Laboratory Medicine, Medical Center, University of Freiburg, Freiburg, Germany; ^6^Faculty of Medicine, University of Freiburg, Freiburg, Germany

**Keywords:** mitochondria, adiponectin, adiponectin receptor, diabetes, heart, proteome

## Abstract

Decreased serum adiponectin levels in type 2 diabetes has been linked to the onset of mitochondrial dysfunction in diabetic complications by impairing AMPK-SIRT1-PGC-1α signaling via impaired adiponectin receptor 1 (AdipoR1) signaling. Here, we aimed to characterize the previously undefined role of disrupted AdipoR1 signaling on the mitochondrial protein composition of cardiac, renal, and hepatic tissues as three organs principally associated with diabetic complications. Comparative proteomics were performed in mitochondria isolated from the heart, kidneys and liver of *Adipor1*^−/−^ mice. A total of 790, 1,573, and 1,833 proteins were identified in cardiac, renal and hepatic mitochondria, respectively. While 121, 98, and 78 proteins were differentially regulated in cardiac, renal, and hepatic tissue of *Adipor*1^−/−^ mice, respectively; only 15 proteins were regulated in the same direction across all investigated tissues. Enrichment analysis of differentially expressed proteins revealed disproportionate representation of proteins involved in oxidative phosphorylation conserved across tissue types. Curated pathway analysis identified HNF4, NRF1, LONP, RICTOR, SURF1, insulin receptor, and PGC-1α as candidate upstream regulators. In high fat-fed non-transgenic mice with obesity and insulin resistance, AdipoR1 gene expression was markedly reduced in heart (−70%), kidney (−80%), and liver (−90%) (all *P* < 0.05) as compared to low fat-fed mice. NRF1 was the only upstream regulator downregulated both in *Adipor*1^−/−^ mice and in high fat-fed mice, suggesting common mechanisms of regulation. Thus, AdipoR1 signaling regulates mitochondrial protein composition across all investigated tissues in a functionally conserved, yet molecularly distinct, manner. The biological significance and potential implications of impaired AdipoR1 signaling are discussed.

## Introduction

Adiponectin is an adipose-derived hormone which comprises 0.01% of all plasma proteins. Despite its known protective functions in the body, which include anti-inflammatory, anti-atherosclerotic and anti-fibrotic effects (1), its primary physiological role may be the ability to improve systemic insulin sensitivity. Its role in regulating glucose homeostasis therefore suggests that the observed decrease in serum levels of adiponectin in non-insulin dependent (type 2) diabetes mellitus (NIDDM) is involved in the pathogenesis of insulin resistance and ultimately NIDDM (2, 3). Impaired adiponectin activity has also been implicated in the development of end-organ diabetic complications including diabetic cardiomyopathy, nephropathy and non-alcoholic steatohepatitis (4–6). Maintaining or restoring intact adiponectin signaling is therefore a promising therapeutic strategy to attenuate these diabetic complications (7).

The molecular effects of adiponectin are principally mediated by its binding to adiponectin receptors 1 and 2 (AdipoR1 and AdipoR2), membrane-bound G protein-coupled receptors which increase the activity of various fundamental intracellular signaling cascades including AMP-activated protein kinase (AMPK), p38 mitogen-activated protein kinase (MAPK), calcium/calmodulin-dependent protein kinase kinase β (CaMKKβ) and peroxisome proliferator-activated receptor α (PPARα) signaling pathways (8–10). A common physiological intersection among these cascades is the regulation of mitochondrial function either by directly regulating substrate oxidation through modulation of enzymatic activity, or by controlling the gene expression of mitochondrial proteins via recruitment of PPARγ coactivator-1α (PGC-1α) (9, 11, 12). Both in skeletal and cardiac muscles, deletion of AdipoR1 in mice impairs mitochondrial function, decreases gene expression of oxidative phosphorylation (OXPHOS) subunits, induces mitochondrial oxidative stress and decreases mitochondrial content, again mediated by disruptions in AMPK/SIRT1/PGC-1α signaling (6, 9).

Mitochondrial dysfunction is a well-known mechanism that contributes both to pathogenesis and complications of type 2 diabetes (13). Mitochondrial defects also contribute to myocardial ischemia reperfusion injury and cardiac hypertrophy, and adiponectin has been shown to attenuate the extent of these cardiac disorders (14, 15). Therefore, these findings suggest that a link may exist between impaired adiponectin action and the resultant mitochondrial dysfunction in the pathogenesis of end-organ diabetic complications. Owing to the incompletely understood effects of impaired adiponectin action on mitochondrial biology, the current study sought to characterize the impact of AdipoR1 signaling on the mitochondrial proteome. Using *Adipor*1^−/−^ mice, we performed comparative mitochondrial proteomics in cardiac, renal, and hepatic tissues to evaluate potential relative contributions of impaired AdipoR1-mediated adiponectin signaling to diabetes-induced mitochondrial defects and proteomic remodeling (16–19).

## Materials and Methods

### Animals

Male mice with global deficiency of AdipoR1 (*Adipor*1^−/−^) and respective C57BL/6J wildtype (WT) littermate controls were investigated at 8 weeks of age, as described previously (6). Mice were housed in individually-ventilated cages with 12 h daylight/dark cycles at 22°C and were fed a laboratory standard chow and had free access to water. Nutritional studies were performed by feeding C57BL/6J mice a high fat diet (Ssniff D12492, 5,15 kcal/g, 20 % protein, 60 % fat, 20 % carbohydrate) or control chow for 12 weeks, starting at the age of 4 weeks. The study conforms to the *Guide for the Care and Use of Laboratory Animals* published by the US National Institutes of Health and was performed after securing approval by the Regierungspräsidium Freiburg (G-16-137).

### Glucose Tolerance Test

Glucose tolerance tests (GTTs) were performed by intraperitoneal injection of glucose at 2 g/kg body weight, using a glucose stock solution of 40% wt/vol D-glucose in 0.9% saline to 12 h overnight fasted mice. Blood sugar was measured using an Accu-Chek Aviva glucometer.

### Quantitative Real-Time PCR

Mice were euthanized using thiopental (200 mg/kg), and hearts, kidneys and livers were removed immediately and snap frozen in liquid nitrogen. Total RNA was isolated from each tissue using TRIzol reagent (Invitrogen, Carlsbad,CA), purified with the RNEasy Kit (Qiagen, Hilden, Germany), and reverse transcribed using the SuperScriptIII Reverse Transcriptase Kit (Invitrogen, Carlsbad, CA) (20). SYBR-green (Invitrogen, Carlsbad, CA) was used as a probe, and amplification was monitored using the CF X96 Real-Time PCR system (Bio Rad, Munich, Germany). Data were normalized to the levels of the invariant transcript α-tubulin and are presented as arbitrary units normalized to wildtype expression levels. Primer sequences are provided in Table S1.

### Proteomic Analysis

Mitochondria were isolated from hearts, kidneys and livers by differential centrifugation as described before (6). Non-mitochondrial contamination was evaluated by performing immunoblots with antibodies recognizing ATP synthase β (mitochondrial membrane marker protein), alpha-tubulin (cytosolic marker protein), and insulin receptor (plasma membrane marker protein), suggesting high purity of mitochondrial isolates (Figure S1). Samples were separated by standard SDS-PAGE on a 12% self-made Bis-Tris gel. Following trypsin digestion, peptides eluted from de-stained gel slices were subjected to mass spectrometric analysis using an LTQ Orbitrap Velos Pro system (Thermo Fisher Scientific) online coupled to an U3000 RSLCnano (Thermo Fisher Scientific) as described previously (21). Employing MaxQuant Vers. 1.5.2.8 (www.maxquant.org) (22), MS/MS spectra were correlated with the UniProt mouse reference proteome set (www.uniprot.org) using the embedded Andromeda (23) search engine. Carbamidomethylated cysteine was considered as a fixed modification along with oxidation (M), and acetylated protein N-termini as variable modifications. For quantitation, LFQ quantitation was enabled with default parameters. False discovery rates were set on both, peptide and protein level to 0.01.

### Bioinformatics and Data Visualization

Data of the proteomics analysis were subjected to functional network and pathway enrichment analyses. Protein-protein network analysis and visualizations were performed using Cytoscape (3.7.2). Literature-curated pathway enrichment was achieved using QIAGEN's Ingenuity Pathway Analysis (IPA®, QIAGEN Redwood City, www.qiagen/ingenuity) on differentially expressed proteins using a low-stringency statistical threshold of *p* < 0.05, followed by Benjamini-Hochberg (BH) *post-hoc* adjustment, as published previously (24). To perform the enrichment, unfiltered differential expression data of all 3 comparisons were first merged into a single data matrix for uploading to IPA via Uniprot identifier. Within IPA, a significance threshold (*P* < 0.05 and |Fold-Change| > 1.5) was applied to each of the 3 comparisons, followed by functional enrichment with results ranked by statistical significance (Table S2). Within this software, pathway analysis was done both on a per tissue basis and as a combined comparison analysis to determine overlapping cross-tissue enrichment of Gene Ontology (GO)-term pathways. For GO-term pathway analysis, enrichment was reported as percent of total genes that annotate to a given pathway (% Enrichment), along with the Bonferroni-Hochberg (B-H)-adjusted *P*-value. Heatmap generation and hierarchical clustering were performed using the *pheatmap* package (1.0.8) within R (3.4.2), and *VennPlex* was used to create the Venn diagrams and determine overlapping gene lists (25). Details of the R coding scripts and other bioinformatics tools used in the current study are published for public use on the following GitHub repository: https://github.com/mepepin/AdipoR1_KO.

### Immunoblotting

Frozen hearts, livers and kidneys were homogenized in modified RIPA buffer containing 50 mM Tris/HCl, 150 mM NaCl, 1% Triton X-100, 0.5% sodium deoxychoilate, 0.1% SDS, 1 mM EDTA, 10 mM sodium fluoride, Protease inhibitor cocktail (Roche Life Sciences, Mannheim, Germany) and Phosphatase inhibitor cocktail 2 and 3 (Sigma-Aldrich, Taufkirchen, Germany), pH 7.5 using an Ultra-Turrax T10 basic homogenizer. Samples were separated by 10% SDS-PAGE and transferred to polyvinylidene fluoride (PVDF) membranes at 250 mA for 2 h and incubated with the following primary antibodies: anti-insulin receptor (1:1000, Cell Signaling Technologies, 3025), anti-NRF1 (1:2000, Abcam, ab175932). Anti-Rabbit IgG (H+L) Fab2 Alexa Fluor (1:5000; Cell Signaling, 4414) and anti-Mouse IgG (H+L) Fab2 Alexa Fluor (1:10000; Cell Signaling, 4408) served as secondary antibody. Detection and quantification of fluorescent bands was performed using the Bio-Rad Western Blot Imager. Loading control was performed using anti-alpha-tubulin (1:2000, Sigma-Aldrich, T9026). For blots investigating levels of MTHFD1L, ATP synthase β and alpha-tubulin performed using isolated mitochondria, the following primary antibodies were used: Anti-MTHFD1L (1:1000, Novus Biologicals NBP2-37864), anti-ATP synthase β (1:2000, BD Biosciences, 612519), anti-alpha-tubulin (1:2000, Sigma-Aldrich, T9026).

### Statistical Analysis

All expression data are reported as mean ± SEM unless otherwise specified. When comparing two groups, significance was determined using a Student's *t*-test using GraphPad Prism 7 software (GraphPad Software, Inc., La Jolla, CA).

## Results

### Global Impact of *Adipor1*^-/-^ on Cardiac, Renal, and Hepatic Mitochondrial Proteome

**Figure 1:**
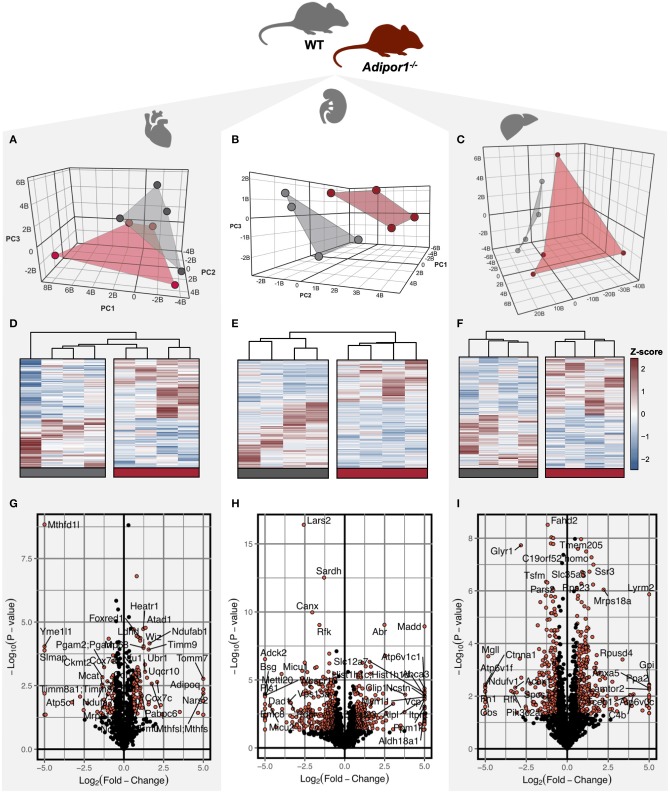
Visualization of differentially regulated proteins in mitochondrial isolates of cardiac, renal, and hepatic tissue of *Adipor1*^−/−^ mice. Unsupervised three-dimensional principal components analysis (PCA) **(A–C)** visualization **(D–F)** of differentially-expressed proteins (P < 0.05), of proteomics data from *Adipor1*^−/−^ mice (red) and wild-type mice (gray) for cardiac, renal, and hepatic tissues; *n* = 4. Volcano plots of differentially regulated proteins in mitochondria of cardiac **(G)**, renal **(H)** and hepatic **(I)** tissue, highlighting those achieving *P* < 0.05 and |Fold-Change| > 1.5.

To determine the impact of disrupted adiponectin receptor signaling on mitochondrial protein composition, a proteomics-based analysis was performed using mitochondria-enriched protein lysate in *Adipor1*^−/−^ relative to age-matched littermate controls. We identified a robust signature of proteins within cardiac (790), renal (1,573), and hepatic (1,833) tissues isolated from both *Adipor1*^−/−^ and littermate controls (Table S3). Of these proteins, 136 (17%) cardiac, 113 (7%) renal, and 93 (5%) hepatic proteins were differentially-expressed in *Adipor1*^−/−^ relative to controls. Unbiased principal components analysis (Figures 1A–C) and hierarchical clustering of statistically-significant (*P* < 0.01) differentially-expressed proteins (Figures 1D–F) revealed a modest separation by genotype for each tissue. Using volcano plot to examine the most robust changes in protein levels in mitochondria of each tissue, we noted that methylenetetrahydrofolate dehydrogenase (NADP^+^ dependent) 1 like protein (MTHFD1L) was the most-robustly decreased (FDR < 0.01) protein in cardiac mitochondria of *Adipor1*^−/−^ mice relative to controls (Figure 1G). By contrast, mitochondrial Leucyl-TRNA Synthetase 2 and Glyoxylate Reductase 1 Homolog were the most robustly suppressed protein in renal and hepatic mitochondrial lysates, respectively (Figures 1H,I).

**Figure 2:**
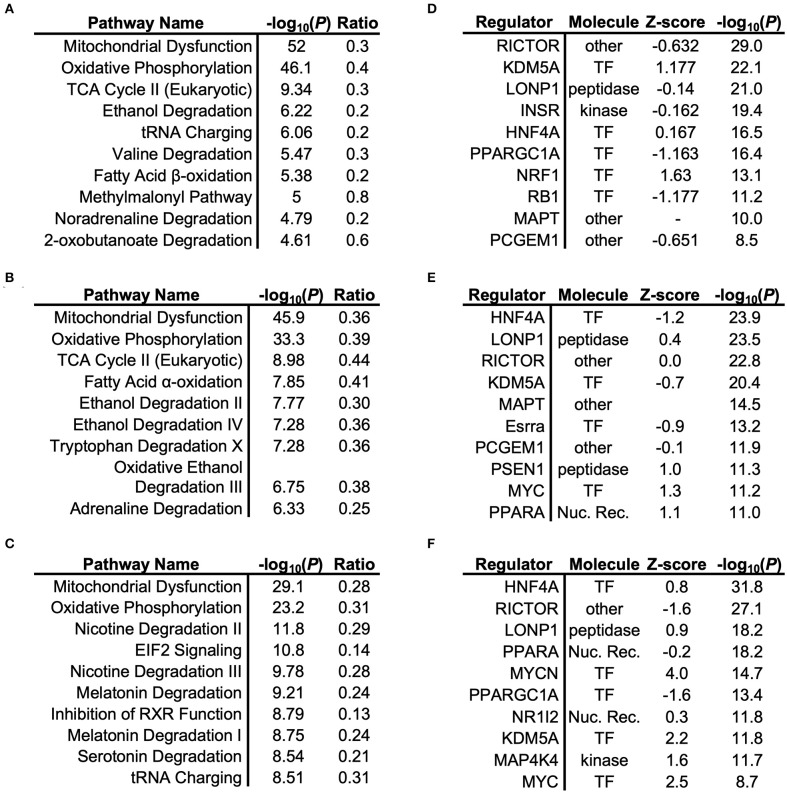
Gene Ontology (GO)-term enrichment analysis of cardiac, renal, and hepatic tissue. Respective pathway enrichment and upstream regulator identification using Ingenuity® comparing proteomics-based analysis of mitochondrial lysates from cardiac **(A,B)**, renal **(C,D)**, and hepatic **(E,F)** tissues from mitochondrial lysates of *Adipor1*^−/−^ mice, expressed relative to WT mice.

To understand the signaling pathways most affected by impaired adiponectin receptor signaling, gene ontology (GO)-term enrichment analysis of differentially-expressed proteins was performed, revealing disproportionate representation of proteins involved in mitochondrial oxidative metabolism, with oxidative phosphorylation, TCA cycle and/or fatty acid oxidation, dependent on the tissue type (Figures 2A–C). Importantly, oxidative phosphorylation was identified as the most regulated specific pathway in each tissue and as the only pathway with significant regulation in every tissue. Predicted upstream regulators most likely responsible for mitochondrial protein changes included the master regulator of mitochondrial biogenesis and previously established AdipoR1 signaling cascade component, Peroxisome proliferator-activated receptor gamma coactivator 1-alpha (PGC-1α) (Figures 2D,F), as well as its downstream transcription factor target, nuclear respiratory factor 1 (NRF1; Figure 2D). Another predicted regulator with high scores for each tissue was Rapamycin-insensitive companion of mammalian target of rapamycin (RICTOR) (Figures 2D–F), which has been previously shown to regulate lipid metabolism in other tissues (26).

### Proteins of Oxidative Phosphorylation Are Disproportionately Suppressed in *Adipor1*^-/-^

**Figure 3:**
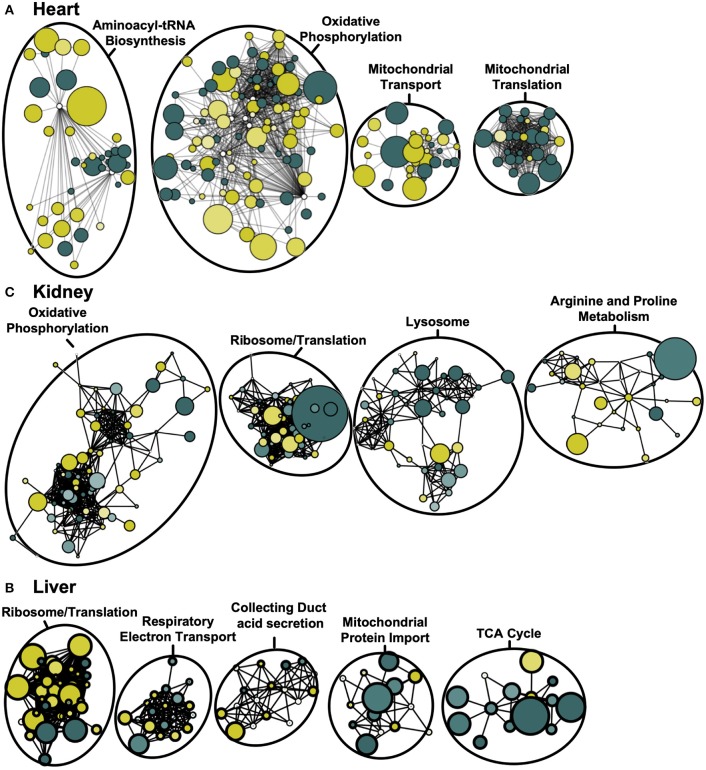
Weighted protein-protein interaction network analysis for cardiac, renal and hepatic mitochondrial proteome. Protein network of differentially-expressed proteins (*P* < 0.05) based on STRING protein interactions (confidence score > 0.80) for heart **(A)**, kidney **(B)**, and liver **(C)**. Functional pathway enrichment terms reported based on the most-enriched on KEGG pathway. Colors represent increased (green) and decreased (yellow) protein levels in *Adipor1*^−/−^ relative to WT mice, with node size inversely proportional to *P*-value.

Although GO-term enrichment is effective in determining which pathways are disproportionately represented among the differentially-expressed mitochondrial proteins, this approach provides limited insight regarding the physical inter-connectedness that may exist among mitochondrial proteins. To begin to appreciate this, we therefore used protein-protein interaction network analyses for each tissue to identify communities of interacting proteins that are together dysregulated by *Adipor1*^−/−^. Using the STRING public database, we generated a weighted matrix of differentially-expressed proteins based on known and predicted physical interactions (Figure 3). Once nodes were ranked by degree of protein-protein interactions, “Oxidative Phosphorylation” was found to be the most dysregulated network both in mitochondria of cardiac and renal tissues (Figures 3A,B). In hepatic tissue, “Ribosome/Translation” represented the most dysregulated functional protein network, although closely followed by “Respiratory Electron Transport” (Figure 3C).

### Systems Biological Proteomics Analysis of *Adipor1*^-/-^ Mice

**Figure 4:**
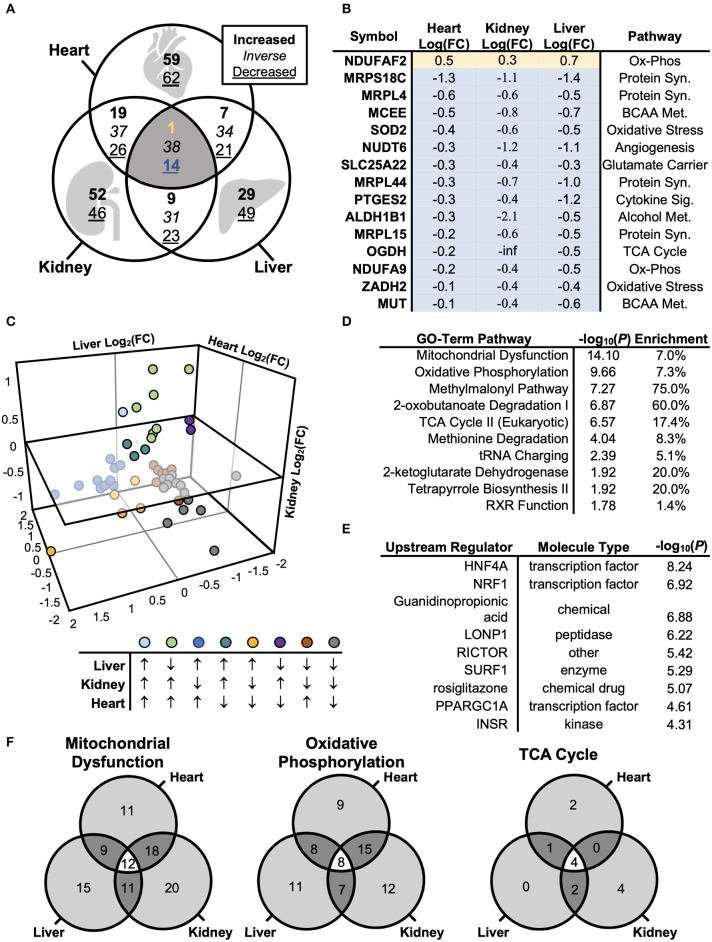
Biological Systems-Based Proteomics Analysis Comparing Cardiac, Renal, and Hepatic Mitochondrial Lysate. **(A)** Venn diagram illustrating differences in protein levels across cardiac, renal, and hepatic mitochondrial isolate from *Adipor1*^−/−^ vs. WT mice (*P* < 0.05). **(B)** Overlapping co-regulated proteins changed within cardiac, renal, and hepatic mitochondrial isolate in *Adipor1*^−/−^ relative to WT mice. **(C)** 3-dimensional scatterplot of the overlapping 53 proteins, demonstrating expression patterns between hepatic, renal, and cardiac tissues. **(D)** GO-Term Pathway Enrichment Analysis via Ingenuity® of co-regulated proteins across all 3 tissue-types. **(E)** Putative upstream regulators via Ingenuity® of co-regulated proteins across all 3 tissue-types. **(F)** Overlap in GO-term enrichment based on tissue-type.

Although adiponectin is known to coordinate energy homeostasis across organ systems (27), the molecular machinery that mediates these signals may differ across tissues. We therefore compared the pattern of protein regulation in mitochondria by disrupted AdipoR1 signaling is consistent or distinct across tissues. We first visualized differentially expressed proteins using a Venn diagram (Figure 4A, Table S4), revealing a modest number of co-expressed mitochondrial proteins. Of the identified proteins, only 15 proteins were regulated in the same direction across all tissue-types (Figure 4B). Interestingly, only one of the 15 differentially co-expressed proteins was increased in the mitochondrial fraction of *Adipor1*^−/−^ mice relative to littermate controls (Figure 4B). In contrast to the 15 commonly regulated proteins, 121, 98, or 78 proteins were regulated specifically in cardiac, renal, or liver tissue, respectively. The number of tissue-specifically dysregulated proteins was always greater than the amount of proteins that were dysregulated in 2-tissue or 3-tissue comparisons (Figure 4A). When comparing differentially regulated proteins of two tissues (e.g., heart and liver), it also became evident that the number of inversely regulated proteins was similar to the amount of up- and downregulated proteins between tissues (e.g., Heart vs. Liver: 34 inversely regulated proteins vs. 28 up-/downregulated proteins). The heterogenous pattern of mitochondrial protein regulation across tissues is also well displayed in a 3-dimensional scatterplot (Figure 4C). These observations indicate that the impact of AdipoR1 deficiency on the mitochondrial protein composition is strongly tissue-specific.

### Proteomic Regulator Analysis and Pathway Enrichment

**Figure 5:**
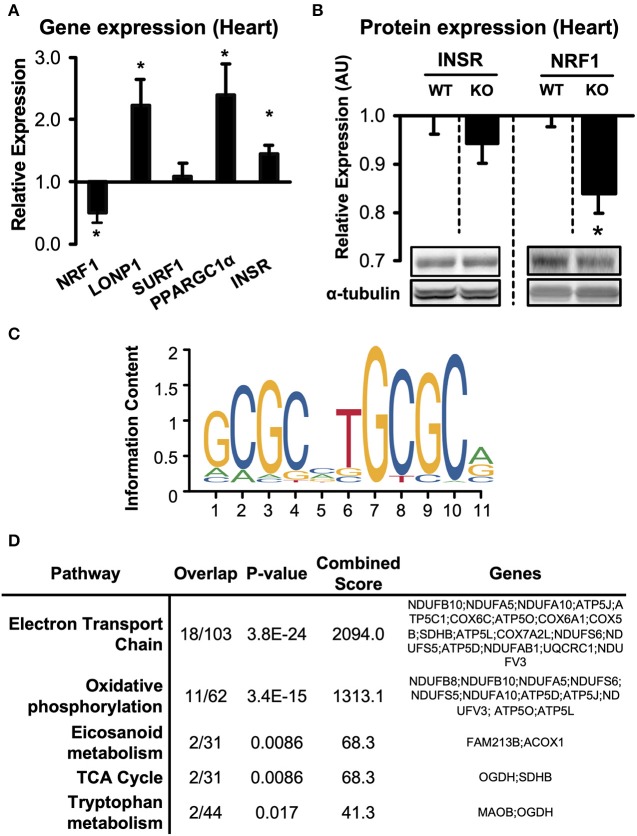
Expression of predicted upstream regulators in hearts of *Adipor1*^−/−^ mice. **(A)** mRNA expression of predicted upstream regulators in hearts of *Adipor1*^−/−^ mice, expressed as fold change relative to expression levels in WT mice; *n* = 6–10. **(B)** Protein levels of NRF1 and INSR in hearts of *Adipor1*^−/−^ and WT mice; *n* = 6. * *P* < 0.05 vs. WT. KO, knock out. **(C)** Position-weighted matrix developed by Morrish et al. (28) to identify genomic locations of putative NRF1 response elements, with **(D)** gene set enrichment using the WikiPathways database of proteins suppressed by *Adipor1*^−/−^ in cardiac tissue (Table S5).

To determine whether adiponectin receptor signaling employs common molecular pathways across tissues, as well as to identify and rank candidate regulators of the proteomic changes, we used the Ingenuity® Pathway Analysis software to populate curated pathways and known molecular networks. This analysis revealed again “Oxidative Phosphorylation” as most enriched GO term (Figure 4D). Hepatocyte Nuclear Factor 4 Alpha (HNF4A) was identified as the most-enriched upstream regulator (*P* < 10^−8^; Figure 5E). Other notable regulators among the top most enriched were NRF1, lon peptidase 1 (LONP), RICTOR, surfeit locus protein 1 (SURF1), PGC-1α, and insulin receptor (INSR) (Figure 4E).

To begin to validate the upstream candidate regulators, we measured mRNA expression of INSR, NRF1, LONP1, SURF1, PGC-1α, and HNF4A in hearts of *Adipor1*^−/−^ mice (Figure 5A). Consistent with the enrichment analysis, four of the five predicted regulators were indeed significantly altered in hearts of *Adipor1*^−/−^ mice compared to wildtype controls. On the protein levels, INSR was unchanged in hearts of *Adipor1*^−/−^ mice compared to WT mice, however protein expression of NRF1 was decreased to similar extents as observed on the mRNA level (Figure 5B), further supporting that Adiponectin receptor 1 signaling is required for NRF1-mediated transcriptional regulation.

To characterize the putative downstream transcriptional effects of NRF1 suppression in hearts of *Adipor1*^−/−^ mice, we used an NRF1 Chromatin Immunoprecipitation (ChIP)-sequencing dataset generated from HepG2 cells by Myers *et al*. (29), and intersected it with putative NRF1 response elements found using the validated NRF1 position-weighted matrix developed by Morrish *et al*. (28) (Figure 5C). This list of genomic positions was then used to identify suppressed proteins possessing at least one NRF1 *motif* in their proximal promoter (within 1.5kB upstream of the TSS) (Table S5). Gene set enrichment analysis identified “electron transport chain” and “oxidative phosphorylation” as common pathways in cardiac tissue (Figure 5D). Taken together, these preliminary observations suggest a downregulation of NRF1-regulated proteins in AdipoR1^−/−^ hearts and implicate NRF1 as a conserved ApidoR1-responsive regulator of mitochondrial respiration.

**Figure 6:**
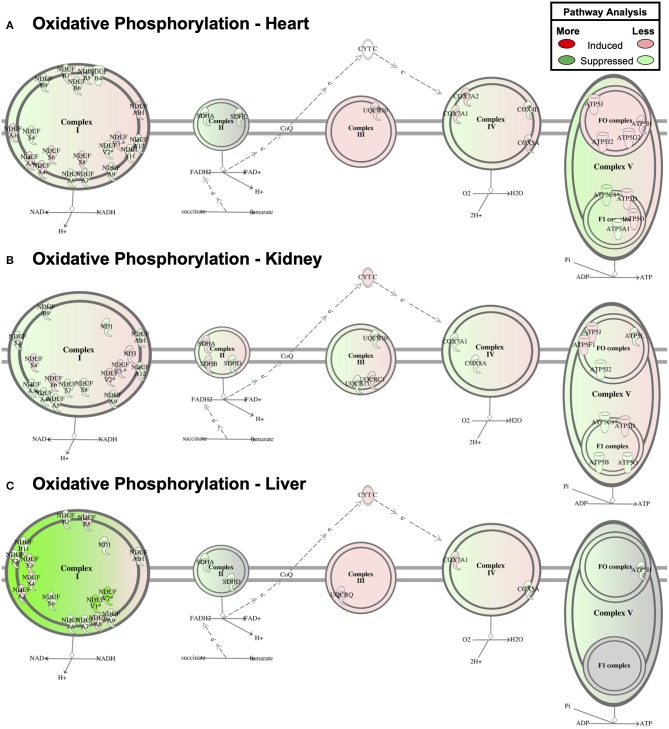
Oxidative Phosphorylation Pathway Enrichment of Cardiac Proteomics. Proteins associated with oxidative phosphorylation were examined for coordinated changes in mitochondrial fractions isolated from cardiac **(A)**, renal **(B)**, and hepatic **(C)** tissue from *Adipor1*^−/−^ and WT mice (*P* < 0.05).

Despite the relative lack of proteomic overlap across the three tissues, pathway enrichment was performed to determine whether this relatively minimal overlap still shows commonly regulated networks or functional pathways (Figure 4F). The three tissues displayed a striking similarity by pathway enrichment, with “Mitochondrial Dysfunction” and “Oxidative Phosphorylation” common among the top 5 pathways. “TCA Cycle” represented another significantly enriched pathway across tissues. However, the differentially expressed proteins responsible for pathway enrichment mainly differed among tissue types (Figure 4F). Figure 6 displays the differentially regulated proteins of the most enriched GO-term among all tissues, i.e., “Oxidative Phosphorylation,” using KEGG pathways. The illustration points out that OXPHOS subunits are primarily less abundant in all tissues, but also emphasizes the heterogeneity in the subunit composition. Taken together, our analysis supports the general conclusion that AdipoR1 signaling regulates mitochondrial protein composition across all investigated tissues in a functionally conserved, yet molecularly distinct, manner.

### High-Fat Diet Suppresses AdipoR1 in a Biologically-Conserved Manner

**Figure 7:**
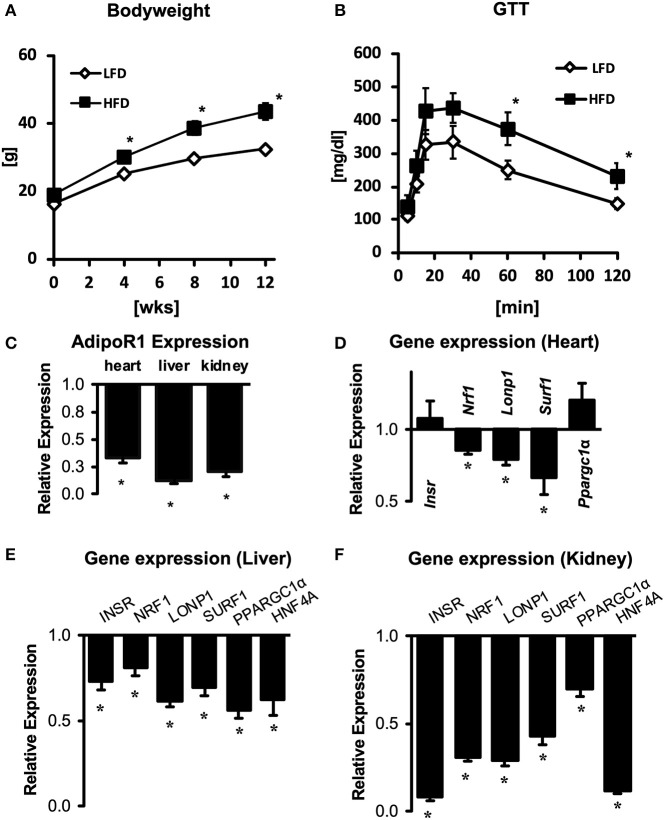
Gene expression of AdipoR1 and predicted upstream regulators in cardiac, renal and hepatic tissue of obese and insulin-resistant mice. Body weight **(A)** and glucose tolerance tests **(B)** of C57BL/6J mice subjected to 12 weeks of LFD or HFD. **(C)** Expression of AdipoR1 mRNA in hearts, livers and kidneys of HFD mice, expressed as fold change relative to expression levels in LFD mice. **(D–F)** Expression of predicted upstream regulators in hearts **(D)**, livers **(E)**, and kidneys **(F)** of HFD mice, expressed as fold change relative to expression levels in LFD mice. *P < 0.05 vs. LFD.

Both mitochondrial dysfunction and impaired adiponectin/AdipoR1 signaling are thought to contribute to tissue damage in diabetes complication organs, including diabetic cardiomyopathy, diabetic nephropathy and non-alcoholic fatty liver disease (30). To evaluate whether impaired AdipoR1 signaling may contribute to mitochondrial dysfunction during the development of insulin resistance and type 2 diabetes, we determined mRNA expression of AdipoR1 in C57BL/6J mice subjected to 12 weeks of low fat (LFD) or high fat diet (HFD). Mice subjected to HFD developed obesity and insulin resistance (Figures 7A,B). Strikingly, we found a marked downregulation of AdipoR1 expression in all tissues, in particular in liver tissue (Figure 7C). Next, we evaluated whether the expression pattern of upstream regulators observed in hearts of *Adipor1*^−/−^ mice may be similarly altered in hearts of HFD-fed mice showing decreased expression of AdipoR1. Only NRF1 was similarly co-regulated in hearts of *Adipor1*^−/−^ mice and HFD-fed mice (Figures 5A, 7D). In kidney and liver tissue of HFD-fed mice, all predicted regulators were clearly downregulated compared to LFD-fed mice, in particular in the kidney, including NRF1 (Figures 7E,F).

## Discussion

NIDDM is a chronic metabolic disease and leading cause of worldwide morbidity today. Although the long-term health risks associated with NIDDM are strongly associated with the degree of glycemic control (31), a multitude of systemic factors have been linked to the eventual development of diabetic complications. Among these circulating molecules, adiponectin has been identified as a key regulator of metabolic homeostasis within liver (2, 32, 33), kidney (34, 35), and cardiac tissues (36). Although impairments in adiponectin signaling are believed to confer mitochondrial toxicity within end-organs affected by longstanding diabetes, the precise molecular intermediates remain unknown, as do the differences across tissue-types. Our use of proteomics-based network analysis of the *Adipor1*^−/−^ model has thus uncovered similarities and differences in mitochondrial protein dynamics among three end-organ tissues most associated with end-organ diabetic complications.

A shared feature prominent in our pathway enrichment analysis was the disproportionate enrichment of oxidative metabolic pathways in *Adipor1*^−/−^ relative to wild-type mice, with “Oxidative phosphorylation” and “TCA Cycle” pathways consistently enriched by differentially expressed proteins (Figure 3). This finding is consistent with prior studies which found that adiponectin-induced AdipoR1-mediated extracellular calcium influx activates the master regulator of mitochondrial biogenesis, PGC-1α, through either calmodulin-dependent protein kinase (CaMK) activity or via AMPK and subsequent SIRT1 activation to promote mitochondrial biogenesis and oxidative capacity in skeletal muscle (9). Our laboratory has previously demonstrated that AdipoR1 deficiency impairs mitochondrial oxidative phosphorylation activity and mitochondrial volume density in the heart (6). Thus, growing evidence supports that adiponectin controls mitochondrial oxidative capacity via AdipoR1-mediated transcriptional regulation of mitochondrial oxidative phosphorylation (OXPHOS). The HFD-feeding associated downregulation of AdipoR1 detected in heart, kidneys and liver suggests that impaired gene expression of OXPHOS proteins in diabetic complications may, at least in part, be driven by impaired AdipoR1/adiponectin signaling, in particular in diabetic cardiomyopathy (37, 38).

In our search for transcriptional regulators capable of potentiating the AdipoR1-mediated effects on metabolism, we found enrichment of NRF1 targets among differentially-expressed proteins by *Adipor1*^−/−^. Furthermore, we found that both *Adipor1*^−/−^ and high-fat feeding were sufficient to suppress mRNA levels of *Nrf1* in cardiac, renal, and hepatic tissues. As an established regulator of mitochondrial biogenesis, NRF1 has been attributed an essential role in the cardioprotective effects of circulating adiponectin via AMPK-PGC-1α-NRF1 metabolic axis, a molecular cascade that may be dysregulated in hearts of murine models of type 2 diabetes (39). Analogous studies of the diabetic liver have found the *db/db* mouse model to display both reduced adiponectin levels and mRNA expression of hepatic *Nrf1*, which is reversed by treatment with recombinant adiponectin (40). Thus, our study links these previously isolated findings to describe how diabetes-associated impairments in adiponectin receptor signaling may trigger transcriptional repression of NRF1 in a biologically-conserved manner, thereby potentially contributing to maladaptive mitochondrial proteomic remodeling.

Of note and in contrast to *Adipor1*^−/−^ hearts, only mRNA levels but not protein levels of NRF1 were reduced in hearts of HFD-fed mice (Figure S2B). Given that AdipoR1 signaling drives NRF1 signaling by increasing NRF1 transcription, this observation may suggest a functional relevance of impaired myocardial AdipoR1 expression in HFD-fed mice, however compensatory post-transcriptional mechanisms may counteract the downregulation of NRF1. One candidate mechanism may be an impaired cardiac ubiquitin-proteasome system in diabetic hearts, potentially leading to less degradation of NRF1 protein (41) Regardless of NRF1 levels, remodeling of the OXPHOS proteome may also very well result from impaired PGC-1α-mediated coactivation of transcription factors such as ERRα or also NRF1 (42). Independent of the specific signaling cascade, some of the differential effects on the mitochondrial proteome composition may also be determined by the differences in tissue-specific *Adipor1* expression levels, thus potentially resulting in varying impact on mitochondrial proteome remodeling if AdipoR1 is absent. Alternatively, the signaling networks with which AdipoR1 signaling may interact or interfere, may be distinct among tissues, thus resulting in differential remodeling of the mitochondrial proteome. Additional studies are required to understand both the determinants and biological significance of the divergent proteomic signatures.

Although our proteomic analysis identified conserved adiponectin-dependent metabolic pathways and central regulators, we also uncovered a divergent network of differentially expressed proteins by *Adipor1*^−/−^ across liver, cardiac, and renal tissues. Despite finding 136 cardiac, 113 renal, and 93 hepatic mitochondrial proteins to be differentially expressed in *Adipor1*^−/−^ relative to controls, only 15 were consistently regulated across the three tissues (Figure 4B). An equivalent degree of phenotypic heterogeneity has been appreciated among other systems-based integrative analyses, wherein tissue-specific regulation is a physiologic requirement for intact hormonal control of a biological system (43, 44). Of particular interest is the tissue-specific remodeling of the OXPHOS proteome, which is the consequence of expression of genes encoded by both the nuclear and mitochondrial genome, followed by incorporation of these proteins via the mitochondrial import apparatus, processes that seem to be specific to the tissue in which mitochondria reside (3, 21, 23, 34). The consequence of such tissue-specific OXPHOS remodeling may be an impairment in ATP regeneration, but also increased leakage of electrons to generate superoxide (45). Extent of ROS, site of ROS production, and nature of ROS (e.g., superoxide, hydrogen peroxide) as a consequence of tissue-specific OXPHOS remodeling in a setting of impaired AdipoR1 signaling may thus determine effects on ROS-dependent signaling and the extent and spatial occurrence of oxidative damage. Indeed, mitochondrial oxidative stress has been observed in hearts of *Adipor1*^−/−^ hearts before (6). Given the incompletely elucidated mechanisms of increased ROS and oxidative stress in diabetes complication organs, OXPHOS remodeling due to impaired AdipoR1 signaling could represent a mechanism that might contribute to increased ROS, a speculation that should be validated experimentally in future studies.

Although we provide strong evidence that AdipoR1 dysregulation negatively impacts mitochondrial pathways, additional experimentation is needed to validate the specific proteins responsible for this enrichment. Immunoblot analysis of MTHFD1L confirmed decreased protein levels in kidney mitochondria of *Adipor1*^−/−^ mice as detected by LC-MS/MS, but failed to confirm a similar decrease in cardiac mitochondria (Figure S3). Other targets were investigated to determine whether mRNA changes of upstream regulators were similarly observed at the protein level in HFD-fed mice, using NRF1 and INSR as exemplary proteins. However, no significant differences in NRF1 could be detected at the protein level in HFD relative to LFD-fed mice (Figure S2A). Discordance between mRNA and protein levels was also observed for INSR, where only liver tissue revealed downregulation of INSR in both mRNA and protein by HFD relative to LFD-feeding (Figure S2B). These findings are either the consequence of false-detection by proteomics, or the inability to detect a true difference with immunoblot analysis based on a myriad of technical considerations such as antibody specificity. Regardless, further experimentation is warranted to validate the specific proteins identified by proteomics.

## Conclusions

AdipoR1 signaling regulates mitochondrial protein composition across three different tissues principally associated with diabetic complications in a functionally conserved, yet molecularly distinct, manner. We speculate that impaired AdipoR1 signaling may causally contribute to features that are typically observed in organs vulnerable to diabetic complications, including impaired ATP synthesis and increased ROS, as a consequence of OXPHOS remodeling. Maintaining AdipoR1 signaling, e.g., by pharmacological activation of AdipoRs using AdipoRon, may represent a useful approach to delay or attenuate the development of specific features observed in diabetes complication organs.

## Data Availability Statement

All data generated or analyzed during this study are included in this published article and are found at the following repository: https://github.com/mepepin/AdipoR1_KO.

## Ethics Statement

The study conforms to the Guide for the Care and Use of Laboratory Animals published by the US National Institutes of Health and was performed after securing approval by the Regierungspräsidium Freiburg (G-16-137).

## Author Contributions

CK, KP, JG, SK, and HB have generated the data. MP, CK, and HB analyzed the data, interpreted the results, and wrote and manuscript. MP, CK, KP, JG, SK, SW, MH, KO, CM, PD, PS, DW, AW, CB, AZ, and HB have read, edited, and approved of the final manuscript.

### Conflict of Interest

The authors declare that the research was conducted in the absence of any commercial or financial relationships that could be construed as a potential conflict of interest.

## Abbreviations

AdipoR1: adiponectin receptor 1
AMPK: AMP-activated protein kinase
CaMK: calcium/calmodulin-dependent protein kinase
CaMKKβ: calcium/calmodulin-dependent protein kinase kinase β
DKD: diabetic kidney disease
Glyr1: glyoxylate Reductase 1 Homolog
HNF4A: hepatic nuclear factor 4 alpha
KO: knock out
LONP1: lon peptidase 1
MAPK: mitogen-activated protein kinase
Mthfd1l: methylenetetrahydrofolate dehydrogenase (NADP^+^ dependent) 1 like protein
mTOR: mammalian target of rapamycin
NAFLD: non-alcoholic fatty liver disease
NASH: non-alcoholic steatohepatitis
NFκB: nuclear factor kappa-light-chain-enhancer of activated B cells
NRF1: nuclear respiratory factor 1
OXPHOS: oxidative phosphorylation
PGC-1α: peroxisome proliferator-activated receptor γ coactivator 1α
PPARα: peroxisome proliferator-activated receptor α
RICTOR: rapamycin-insensitive mTOR
ROS: reactive oxygen species
SIRT1: sirtuin 1
STAT3: signal transducer and activator of transcription 3
SURF1: surfeit locus protein 1.
