# Supplementary material for: Dysregulation of the Mitochondrial Proteome Occurs in Mice Lacking Adiponectin Receptor 1
Source: Front Endocrinol (Lausanne). 2019 Dec 13;10:872. doi: 10.3389/fendo.2019.00872 (PMC6923683; doi:10.3389/fendo.2019.00872)
Supplement: Supplementary file 1 [file Table_1.DOCX]

**Table S1.** Sequences of forward and reverse primers used for RT-PCR.

**Gene Primer Primer Sequence (5´-3`)**

Rps16 Forward TGCTGGTGTGGATATTCGGG

Reverse CCTTGAGATGGGCTTATCGG

AdipoR1 Forward GTCCACCATCACAGGAAGA

Reverse GCTTGCCCTTCTCCTCCA

NRF1 Forward CTTCAGAACTGCCAACCACA

Reverse GCTTCTGCCAGTGATGCTAC

LONP1 Forward GCTGCCACTGACACATCCAAG

Reverse GCGATGATATCCCGAATGGTC

SURF1 Forward CTGGCAGACTATCCTCAACTG

Reverse CTGTCTGGTCTCAGGATTCAC

PPARGC-1α Forward GTAAATCTGCGGGATGATGG

Reverse AGCAGGGTCAAAATCGTCTG

INSR Forward TGGTCCTTTGGGAAATCACT

Reverse ATCCTTGAGCAGGTTGACGA

HNF4α Forward GGATATGGCCGACTACAGCG

Reverse TGTGGTTCTTCCTCACGCTC

Abbreviations: s16rRNA, 16S ribosomal RNA; AdipoR1, adiponectin receptor 1; Nrf1, nuclear respiratory factor 1; LONP1, mitochondrial lon peptidase 1; SURF1, surfeit gene 1; PPARGC-1α, peroxisome proliferator-activated receptor gamma coactivator 1 alpha; INSR, insulin receptor; HNF4A, hepatic nuclear factor 4 alpha
