# Supplementary figures and images for: Dysregulation of the Mitochondrial Proteome Occurs in Mice Lacking Adiponectin Receptor 1
Source: Front Endocrinol (Lausanne). 2019 Dec 13;10:872. doi: 10.3389/fendo.2019.00872 (PMC6923683; doi:10.3389/fendo.2019.00872)

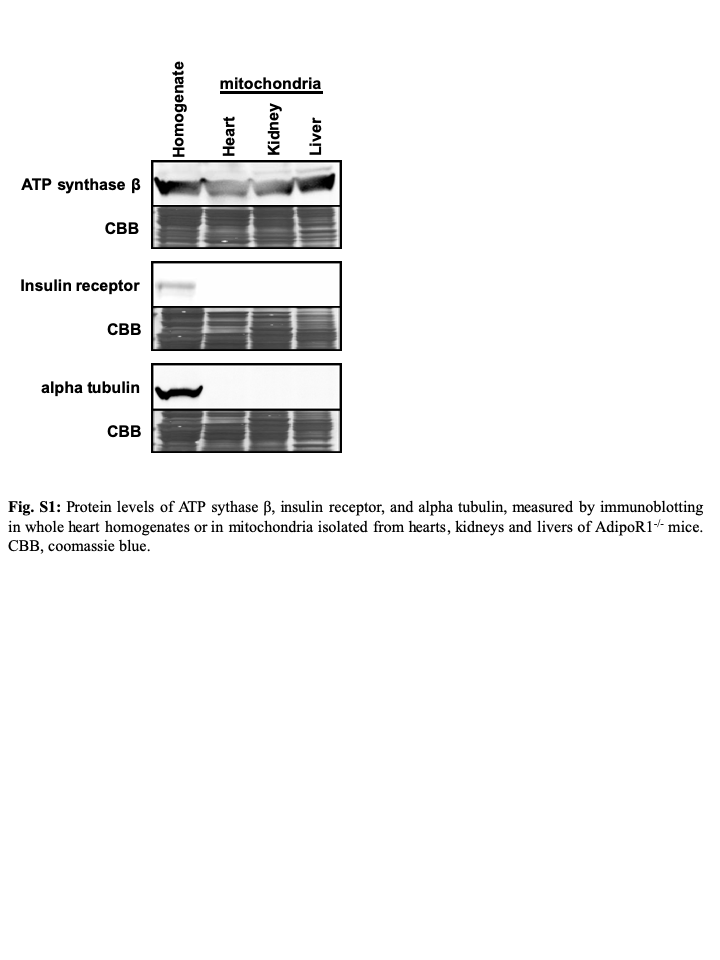

Supplement: Supplementary file 6 [file Image_1.TIFF]

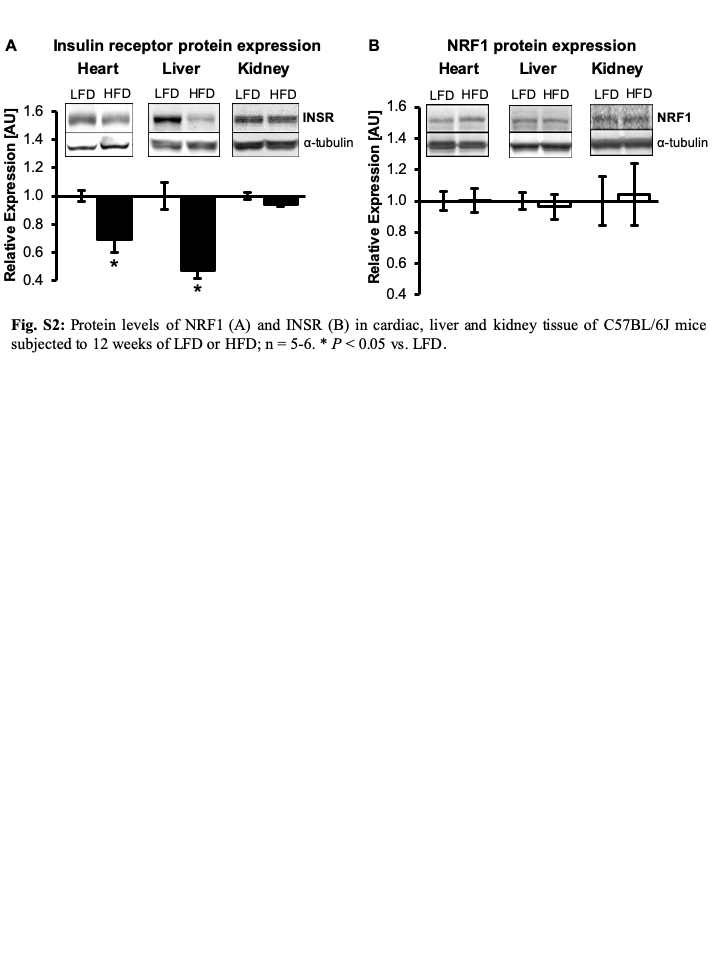

Supplement: Supplementary file 7 [file Image_2.TIFF]

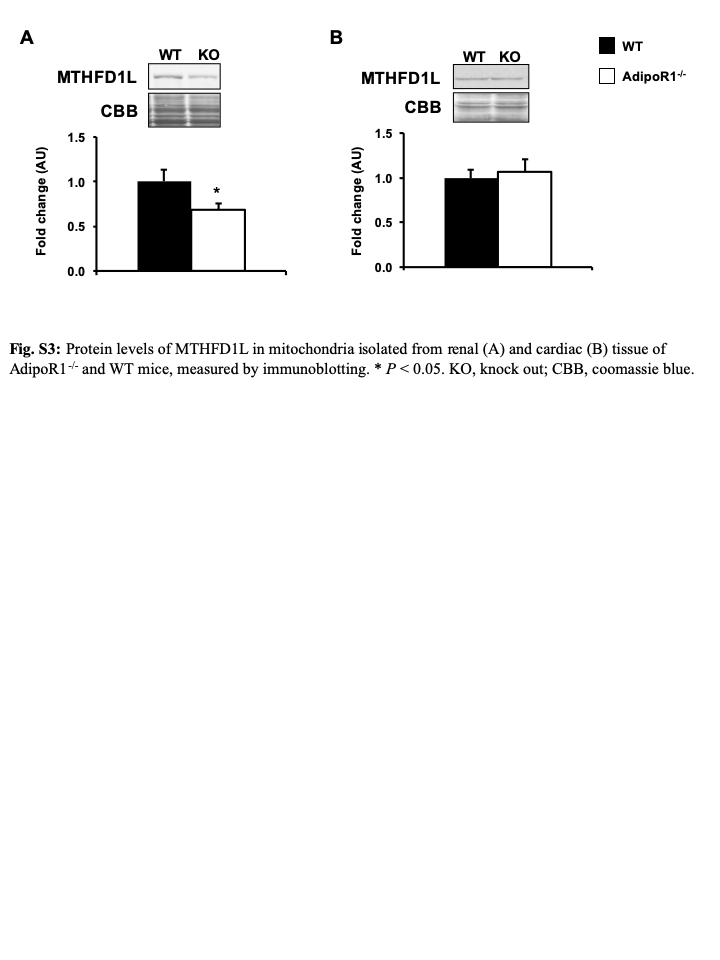

Supplement: Supplementary file 8 [file Image_3.TIFF]
